# Mechanism of Cancer Growth Suppression of Alpha-Fetoprotein Derived Growth Inhibitory Peptides (GIP): Comparison of GIP-34 *versus* GIP-8 (AFPep). Updates and Prospects

**DOI:** 10.3390/cancers3022709

**Published:** 2011-06-20

**Authors:** Gerald J. Mizejewski

**Affiliations:** Division of Translational Medicine, Wadsworth Center, New York State Department of Health, Empire State Plaza, Albany, NY 12201, USA; E-Mail: mizejew@wadsworth.org; Tel.: +1-518-486-5900; Fax: +1-518-402-5002

**Keywords:** alpha-fetoprotein, cell cycle, AFP receptor, AFPep, cancer cells, AFP-derived GIP, Growth Inhibitory Peptide, breast cancer

## Abstract

The Alpha-fetoprotein (AFP) derived Growth Inhibitory Peptide (GIP) is a 34-amino acid segment of the full-length human AFP molecule that inhibits tumor growth and metastasis. The GIP-34 and its carboxy-terminal 8-mer segment, termed GIP-8, were found to be effective as anti-cancer therapeutic peptides against nine different human cancer types. Following the uptake of GIP-34 and GIP-8 into the cell cytoplasm, each follows slightly different signal transduction cascades en route to inhibitory pathways of tumor cell growth and proliferation. The parallel mechanisms of action of GIP-34 *versus* GIP-8 are demonstrated to involve interference of signaling transduction cascades that ultimately result in: (1) cell cycle S-phase/G2-phase arrest; (2) prevention of cyclin inhibitor degradation; (3) protection of p53 from inactivation by phosphorylation; and (4) blockage of K^+^ ion channels opened by estradiol and epidermal growth factor (EGF). The overall mechanisms of action of both peptides are discussed in light of their differing modes of cell attachment and uptake fortified by RNA microarray analysis and electrophysiologic measurements of cell membrane conductance and resistance. As a chemotherapeutic adjunct, the GIPs could potentially aid in alleviating the negative side effects of: (1) tamoxifen resistance, uterine hyperplasia/cancer, and blood clotting; (2) Herceptin antibody resistance and cardiac (arrest) arrhythmias; and (3) doxorubicin's bystander cell toxicity.

## 1. Introduction

**Figure 1.**
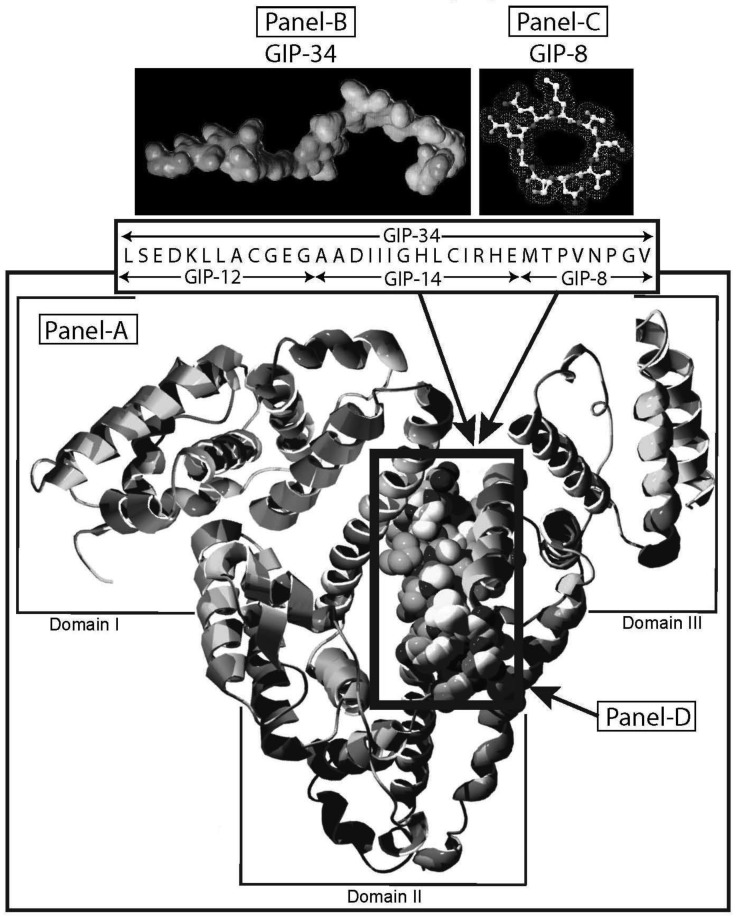
(**A**). A three-dimensional v-shaped helix/ribbon computer model of human alpha-fetoprotein (HAFP) is displayed. GIP-34 amino acid buried segment (**D**) is shown in the black boxed configuration. Minimal energy computer model of GIP-34 and GIP-8 and their amino acid sequences are displayed above the v-shaped HAFP model (**B** and **C**).

Human alpha-fetoprotein (HAFP) is tumor-associated fetal protein, termed an oncofetal protein, consisting of 609 amino acids (AAs) including a 19 amino acid (AA) signal sequence [1-3]. HAFP has been shown to be a growth enhancing factor in its circulating, compact-folded, native full length configuration in studies of both fetal and tumor cell growth and proliferation studies (Figure 1A) [4-6]. Having served as a serum biomarker for cancers of the liver, gonads, and gastrointestinal tract, HAFP is now being investigated as an activator of cell surface receptors as well as a regulator of cytoplasmic transcription factors involved in signaling pathways [7-9]. When present in stress and shock environments, full-length HAFP undergoes a conformational change which temporarily converts the growth enhancing oncofetal protein to a growth inhibitory form referred to as “transformed AFP” (tAFP) [10,11]. The peptide sequence of the transforming site on AFP has been identified and found to be composed of a 34-AA portion of the HAFP molecule, which serves as a growth inhibitory segment (Figure 1) [12]. This 34-AA stretch has been synthesized as a free peptide fragment and termed the “Growth Inhibitory Peptide” (GIP); it has since undergone extensive biological and biochemical characterization [12-15].

The GIP fragment derived from HAFP has been reported to be a negative regulator of both fetal and cancerous growth and is composed of three contiguous bioactive subfragments consisting of 12 AAs (GIP-12), 14 AAs (GIP-14), and 8 AAs (GIP-8) [11,16-19] (Figure 1B, C). Although all three peptide fragments display bioactivity in various *in vitro* and *in vivo* growth models, GIP-34 and GIP-8 have consistently demonstrated anti-cancer growth activity [19-21]. While GIP-8 appears to function largely in estrogen (E)-dependent cancers, GIP-34 was found to inhibit both E-dependent and non-E-dependent (basal) cancer growth [22]. Both the GIP-34 and GIP-8 segments were first discovered by the author in 1993 using uterine growth and cancer cell models [12]. Since then, GIP-8 has been referred to “AFPep” in a series of publications from the several investigative groups [21,23]. These various investigative teams had initiated studies on the 8-mer peptide which have since confirmed and extended the work of Mizejewski *et al.* and extended the utility of GIP-8 (AFPep) as an anti-cancer agent [23,24]. Although some clues to the functional roles of both GIP-34 and GIP-8 have been sporadically reported, the mechanism of anti-cancer growth of the two AFP-derived peptides has yet to be clarified. Therefore, the objectives of the present report will be four-fold. First, the characteristics, properties, and traits of GIP-34 and GIP-8 will be reviewed and updated to bring the reader current with the biomedical literature. Second, published reports contributing to the understanding of the mechanism of action of the two peptides will be discussed in order of their disclosures and advancements. Third, the mechanism of action of each peptide will be discussed and presented in a peptide-to-peptide comparison. The GIP comparison will start with the initial binding of the peptide to the cell surface and extend to the cytoplasmic destinations and/or subcellular targeting of the individual peptides. Finally, a discussion of the advantages and disadvantages in the use of each peptide will be presented. The tables and figures demonstrate how each peptide activity contributes toward clarifying their mechanism of action of cancer growth suppression.

## 2. GIP-34: Properties, Traits, and Biological Activities

**Figure 2.**
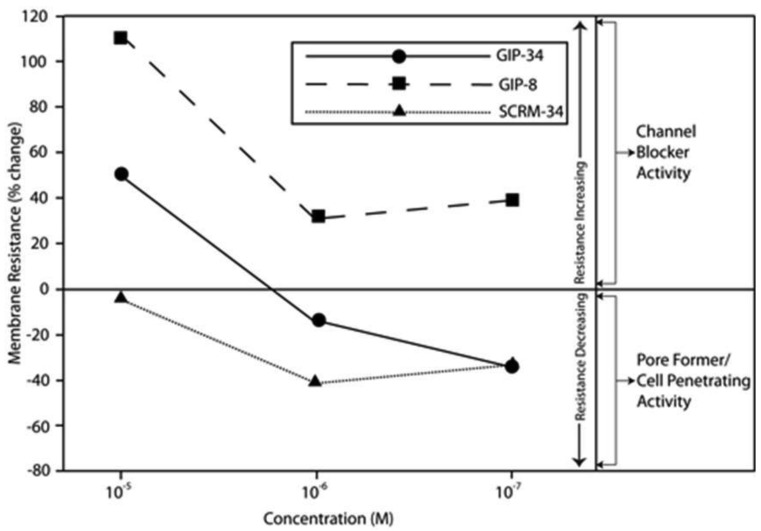
Growth Inhibitory Peptides (GIP-34, GIP-8, and a 34-mer scrambled (SCRM) peptide were evaluated for acute effects on cell membrane potential (current) and membrane resistance in cultured human breast cancer cells. MCF-7 cells were impaled *in vivo* with sharp microelectrodes under inverted phase contrast microscopy.

**Figure 3.**
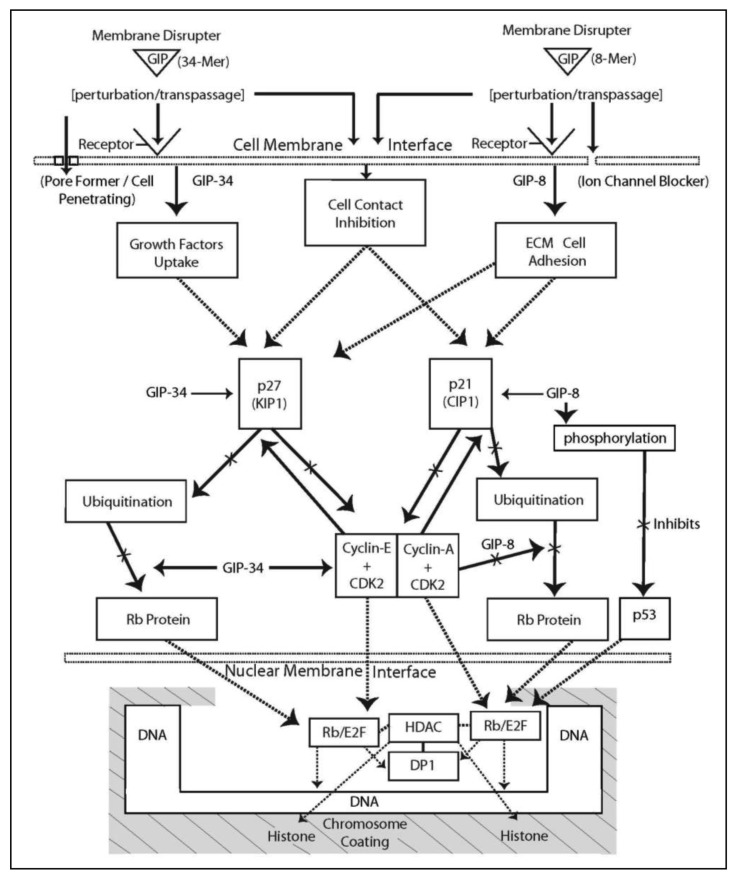
A flow diagram showing the proposed mechanism of growth inhibition of cancer cells by GIP-34 (left side) and GIP-8 (right side). Solid black-line arrows indicate pathways verified by direct evidence; the dash-line arrows represent hypothetical, proposed, or published pathways.

The biological activities of GIP are cataloged and listed in chronological order in Table 1. The secondary structure analysis of GIP-34 revealed an amphipathic peptide consisting of 45% beta sheets and turns, 45% random coil (disorder) and 10% alpha-helix [13-15,25]. GIP-34 displays a carboxyl-terminal type-I reverse beta turn as does the 8-mer peptide [26,27]. This type of beta-turn has been demonstrated to enhance the biological activity of ligand binding to cell surface receptors; such studies revealed that this receptor topology is known to preferentially accommodate the beta-turn in ligand-to-receptor binding kinetics [26]. GIP-34 has been shown to bind to the plasma membrane of human MCF-7 breast cancer cells and concomitant pulse-chase experiments indicated that this contact resulted in rapid cell internalization of the peptide within 1–5 min [19,28]. The peptide undergoes subsequent transmembrane passage into the cytosol and within 1.0 h the peptide is observed in a diffusely scattered pattern throughout the cytosol; by 2.0 h the peptide is trafficked to the perinuclear region of the endoplasmic reticulum, an area which immediately surrounds the nucleus [19]. In addition, evidence obtained from electrophysiologic Sharp microelectrode whole cell recordings of MCF-7 tumor cells was obtained using glass micropipettes filled with 3 M potassium acetate and 0.1 M potassium chloride with an inserted chloridized silver wire. Membrane potential was recorded at room temperature with an Axoclamp 2A (Axon Instruments) multifunction amplifier in constant current mode. Membrane resistance was determined by passing 70 msec 200 pA hyperpolarizing constant current square-wave pulses at 280 msec intervals, measuring the corresponding voltage deflections and applying Ohm's law. *In vivo* recordings indicated that GIP-34, at 10^−6^ M and lower concentrations serves as a cell membrane pore forming/cell penetrating peptide coincident with decreased cell membrane resistance; at high peptide concentrations (10^−5^ M and greater) GIP-34 acts as a channel blocker coincident with increased cell membrane resistance [28] (Figure 2). Patch clamp experiments also confirmed that low concentrations of GIP produced decreased membrane resistance (pore-forming). The pore-forming/cell penetrating molecules have been shown to be amphipathic peptides of >20 AAs and resemble the antimicrobial peptides which are discussed below [29]. In contrast, a channel blocker is a drug or peptide that interacts at the plasma membrane in juxtaposition to an ion channel nestled among a macromolecular cluster of signaling proteins (signalplex) on the inner side of the cell membrane [30]. Thus, GIP-34 can gain entrance into cancer cells by at least two pathways namely, (a) receptor-mediated endocystosis and (b) pore-forming/cell penetration (Figure 3).

By means of a global RNA microarray analysis, GIP-34 was found to down-regulate the RNA of outward flux K^+^ ion channels that determine cell membrane potential and conductance in MCF-7 cells (Table 2) [28]. The voltage range (−30 to −45 mVolts) representing the MCF-7 cell membrane K^+^ ion channel depolarization has been previously studied in MCF-7 cells and was shown to correlate with S-Phase events of the cell cycle [31]. The line graphs displayed in Figure 2 indicate that GIP-34 can serve either as a pore forming/cell penetrating peptide or as a channel blocker depending on peptide concentration, while GIP-8 can act only as a channel blocker [28]. Once within the cytosol, GIP-34 has been demonstrated by microarray analysis, to down-regulate Cyclin-E, SKP2, and various cell cycle RNA transcripts of proteins which prevent Cyclin-E/Cdc2 progression of G1 to S-phase; it also blocked ubiquitin-initiated degradation of cyclin inhibitors such as p27 KIP and p21 CIP as shown by Western blots [32,33].

Previous reports have documented that GIP-34 is able to suppress tumor cell growth and proliferation in various rodent and human cancer cell models [34-36]. Moreover, the biological activity of GIP-34 is dependent on its molar concentration and its oligomeric state, that is, whether it is in a linear or cyclic configuration [34]. While the more labile linear version can form trimers, the cyclic version behaves like a dimer; the linear form is growth inhibitory at 10^−4^ to 10^−6^M, while the cyclic form is inhibitory at 10^−7^ to 10^−10^M concentrations [34,35]. In cell adhesion studies, the 34-mer peptide inhibited tumor cell attachment to a variety of extracellular matrix (ECM) proteins, some of which serve as basement membrane and cellular anchor constituents such as fibronectin (FBN), vitronectin (Vn), collagen (Col), thrombospondin (TBS), fibrinogen (FN), and laminin (LAM) [22,34]. In assays employing activated human platelet suspensions, GIP-34 was able to block 90%-95% of all stages of the platelet aggregation process [34,36]. In angiogenesis assays, the 34-mer peptide inhibited 95% of blood vessel formation in both chick embryo and in cancer cell assays [36]. The disruption by GIP-34 of cell surface activities such as tumor cell adhesion, cell pseudopodial extensions, platelet aggregation, and cell agglutination was shown to severely disturb, impair, and disable the ability of tumor cells to transduce signals, spread, adhere, and metastasize [34,36]. It is for this reason that GIP-34 has been described as a cell membrane disruptive agent.

Concerning the immune response, reports have shown that the GIP-34 and its subfragments can both regulate and invoke an immune response of the cellular type (T-cells, dendritic cells, *etc.).* However, a humoral immune response to GIP-34 or its subfragments have yet to be reported. Overall, GIP-34 has been found to produce an enhancement of cellular immune function when tested in a Con-A stimulated blast transformation assay employing splenic T- and β-cells *in vitro* [22,36]. The proliferation response was measured using H^−3^thymidine incorporation and indicated that GIP-34 enhanced lectin-induced blast cell transformation, but did not suppress cellular immune function. Both the middle (GIP-14) and terminal fragments (GIP-8) have been determined to be antigenic epitopes for the activation of dendritic cells and T-lymphocytes [37-39]. Several other research groups have confirmed that not all HAFP-specific T-cell clones have been deleted from the immunoregulatory repertoire in humans during fetal development; thus, antigenic sites persist in adult life which are recognized in both murine and human T-cell models [40-44]. Computer generated HAFP AA sequences consisting of 9–10 AAs in length and comprising 74 candidates peptides, were synthesized and screened by lymphocyte assays in which 14 major histocompatibility (MHC) antigenic sites of the HLA-A epitope type were identified [37,38]. Interestingly, two of the selected 9 AA sequences corresponded to sequences within GIP-34, namely, the middle and terminal sequences CIRHEMTPV and PVNPGVGQC. Each of two peptide segments proved capable of producing specific T-cell activation and inducing cytokine secretion *in vitro* from normal HLA-A-0201 human donor lymphocytes. The GIP peptide epitopes recognized HLA-A-0201/AFP primed tumor cells in cytotoxicity assays and induced interferon (IFN-alpha) cytokine production and secretion assays [22,36]. Thus, AFP peptide-specific primed T-cells were also identified in spleens of mice immunized with dendritic (antigen-presenting) cells transduced with an AFP-expressing adenovirus system [39,42]. Thus, two juxtaposed and overlapping subfragments of GIP-34 can serve as antigenic epitopes that are immunogenic and would be capable of inducing T-cell immune responses during the course of peptide anti-cancer therapy. To date, only enhancement of the cellular immune response has been reported following use of GIP *in vitro* and *in vivo*; theoretically, this could aid in bolstering the immune defenses of a patient under treatment with GIPs. Moreover, immunotherapeutic treatment of human patients receiving AFP-derived peptides in ongoing clinical Phase-1 trials has not reported any detrimental side-effects to date [40,41]. Thus, human clinical trial results show only beneficial effects of HAFP-derived peptides in patients undergoing hepatoma immunotherapy.

Regarding a receptor for GIP-34, a recent collaborative study has reported efforts to identify a GIP receptor involved in cancer cell targeting [45,46]. In a report of an international collaborative study [45], both GIP-34 and GIP-8 were clearly bound at the cell surface and taken up by MCF-7 and other cancer cells. In addition, GIP-34 was shown in that study to suppress the *in vitro* growth of multiple tumors including T-cell lymphomas, hepatomas, and ductal and glandular breast carcinomas. It was found that GIP-34 produced greater tumor growth suppression than did tamoxifen alone in comparison studies. It had previously been demonstrated that GIP-34 suppressed the *in vivo* growth of tamoxifen-resistant GI-101 human breast cancer xenografts in mice [35]. In that collaborative report, it corroborated the observation that radiolabeled GIP-34 could bind to cancer cells being localized in rodent mammary tumors *in vivo* at 24 h post-IV-injection. By means of cross-linking and pull-down assays utilizing tumor cell lysates, it was reported that radiolabeled GIP-34 bound to an intrinsic mammary tumor cell protein with an apparent molecular mass of 30 kD [45]. A similar study included in that collaborative report showed that GIP-34 conjugated to fluorescent nanobeads was observed both at the cell surface and within the cytosol of MCF-7 cultured tumor cells. Again using cross-linking methods, a separate author demonstrated that GIP-34 bound to a 15 kD protein obtained from MCF-7 cell lysates. Thus, two separate groups of investigators from the same collaborative report showed that GIP-34 bound to two different protein moieties (possibly oligomers) in breast tumor cells displaying masses of 15 kD and 30 kD which indicated the presence of GIP receptors or binding proteins [45]. While searching for a receptor for GIP-34 and GIP-8 in a separate report, Mizejewski showed that neither GIP-34 nor GIP-8 bound to an AFP receptor which was previously described by Moro [36]. A family of proteins termed scavenger receptors has recently been proposed as a candidate receptor protein family for full-length AFP due to their pattern recognition ligand binding properties [46].

## 3. The GIP-8 Segment: Properties, Traits and Biological Activities

The biological activities of GIP-8 are cataloged and listed in chronological order in Table 1. The GIP-8 segment (also called AFPep, P149c, P447, P472-2) is an 8-amino acid peptide first reported by the author (GJM) in 1995 as an anti-cancer and anti-growth fragment derived from the 34-mer GIP segment [12,22,47]. A trypsin digest of the GIP-34 peptide yielded an 8–9 amino acid fragment which was found to possess the E-dependent anti-cancer growth properties of the original 34-mer peptide. During the next several years, GIP-8 was synthesized, characterized, and bioassayed by the author in a series of studies confirming that GIP-8 could both prevent growth and suppress proliferation of E-dependent breast cancer and immature uterine cells [22]. Interestingly, the identical suppression of E-induced immature uterine growth demonstrated by GIP-34 was also found to reside in the GIP-8 subfragment [18]; however, only GIP-34 itself and certain subfragments were found to bind both the human estrogen receptor (ER) and estradiol, while the GIP-8 alone did not bind these moieties [11]. In that same time period, Mesfin *et al.* succeeded in cyclizing the GIP-8 by the addition of a terminal amino acid (Gln or Asn) to form a 9-mer peptide and substituting hydroxyproline for proline in the original sequence [23,48]. Their studies subsequently confirmed that the cyclized GIP-8 maintained the same E-dependent anti-cancer growth properties as the original linear 8-mer version. Following those studies, Bennett *et al.* reported that the cyclized GIP-8 (AFPep) prevented the growth of tamoxifen in MCF-7 human breast cancer xenografts and in tamoxifen-resistant breast cancer xenografts in mice and suppressed the uterotrophic hyperplasia produced by tamoxifen in immature mice [21,24]. Mizejewski and MacColl then demonstrated that the biological activity of GIP-34 *versus* GIP-8 differed in that the latter peptide could only suppress E-stimulated normal uterine and tumor growth above that of basal growth, while GIP-34 could inhibit both E-dependent growth and basal growth irrespective of estrogen stimulation [19]. In contrast to published reports [21,23,24], data obtained from other laboratories employing GIP-8 have shown the 8-mer peptide is capable of both anti-cancer and anti-growth activities in E-supported growth and in non-E-supported basal growth in cancer types, such as glioblastomas, kidney tumors, and mammary ascites tumors [20,22,36]. Mizejewski *et al.* later established that GIP-8 could inhibit the basal growth of kidney tumors by 38%, MCF-7 breast cancer E-dependent growth by 45%, and E-induced immature uterine growth by 42% in a series of studies employing dose response curves [11,18,19,25].

It was a study by Defreest *et al.* that first demonstrated that the pharmacophore of the GIP-8 was located in the middle-portion of the 8-mer peptide showing that only some of the 8 amino acids were essential for peptide growth inhibitory function, while others were neutral [49,50]. The results from this group established that the essential AAs were Glu, Val, Asn, Pro, and Gly, and that Val could be substituted by Ile, Leu, or Ser. In a report by Shields *et al.*, it was revealed that the secondary structure of GIP-8 consisted of a random coil containing a reverse beta-turn, a structure known to bind and activate cell surface receptors linked to G-proteins [26,27]. It was further disclosed that the beta-turn was located in a tetra-amino acid sequence of the 8-mer, namely, Thr-Pro-Val-Asn (TPVN), but not in the Pro-Val-Asn-Pro (PVNP) sequence which is involved as in the activation of serine/threonine Src-kinases [51,52]. It is of interest that the TPVN sequence is a known motif for peptide binding to PDZ-containing proteins and non-selective cation (K^+^, Ca^++^) channels termed Transient Receptor Potential (TRP) channels [53]. Muehleman *et al.* then reported that GIP-8 prevented tumor cell adhesion to ECM proteins by 56% and could itself adhere to many such proteins including TBS, COL, FBN, FN, and Lam [34,36]. Parikh *et al.* then posited that AFPep was instrumental in the prevention and decreased incidence of nitrosourea-inducted breast tumors in rats while confirming that GIP-8 could inhibit the E-supported growth of MCF-7 breast cancer in murine xenografts [54,55]. Bennett *et al.* then presented evidence that GIP-8 was orally-active in the induction of the nitrosourea-induced rat breast tumors and that GIP-8 interfered with the phosphorylation of the human estrogen receptor (ER) at serine-118 and also with phosphorylation of p53 at serine-15 and serine-392 [24]. The phosphorylation of p53 was previously reported to inactivate this tumor suppressor in transformed cell cultures [56].

Subsequently, evidence was forwarded by Andersen *et al.* confirming that GIP-8 reduced uterine hyperplasia while simultaneously increasing the anti-tumor effect of tamoxifen [21,55,57,58]. In these later studies, it was shown that administering low doses of both GIP-8 and tamoxifen reduced tamoxifen (TAM) toxicity and overcame TAM resistance in MCF-7 cells which had been selected for tamoxifen-resistance. In normal cycling rats, it was further found that GIP-8 did not disrupt the estrus cycle nor did it affect fertility of the female [58]. Prior to that, Butterstein *et al.* had related that the linear GIP-8 reduced E-induced toxicity by 37% in pregnant mice, while protecting mothers (dams) against reduced liter sizes produced in E-induced toxic pregnancies [17].

In a series of sequential studies, Torres and Sierralta *et al.* reported that GIP-8 not only inhibited E-induced growth but also suppressed epidermal growth factor (EGF)-induced growth by 40% in cultured ZR75 breast cancer cells [59-61,62]. In these pursuits, it was determined that the 8-mer peptide interfered with regulation of the MAPK-kinases activated by c-erbB2 (EFGR2) receptors. However, GIP-8 seemed to have no effect on MMP-2, MMP-9, and heparin-binding EGF cancer cell surface shedding, all of which are prerequisites for metastasis. Following that study, Sierralta *et al.* showed that GIP-8 had no effect on apoptosis and did not alter E-cadherin levels, but did modulate the cytosolic levels of p21 CIP which act to hinder cell cycle transition of S to G2 phase [63]. Finally, it was demonstrated that GIP-8 could be conjugated to doxorubicin (DOX) in order to improve chemotherapeutic drug delivery and increase toxicity of the chemodrug. The GIP-DOX conjugate exhibited cytotoxic efficacy that matched DOX alone, showed specificity for binding to cancer cells, and enhanced peptide/DOX delivery across cancer cell membranes and into the cytoplasm [45].

In general, both the linear and cyclic versions of GIP-8 have demonstrated growth suppression in multiple different cancer cell assays [21,23,36]. The growth suppressive properties of GIP-8 appears to be initiated as a cell surface phenomenon associated with hydrophobic compartments of the bi-lipid plasma membrane; this was followed by peptide interaction with a receptor or an ion channel complex [36,45,64]. Such data indicate that GIP-8 can serve as a plasma membrane interactive agent that may block ion channels and interfere with cell surface-induced signal transduction pathways involved in tumor growth, progression, and metastasis.

## 4. Proposed Mechanism of Action: GIP-34

The mechanism of action of GIP-34 being proposed is based on experimental data accumulated from multiple laboratories over the last 17 years (Table 1). The data was obtained from *in vitro* cell culture, *in vivo* xenografts and isografts, RNA microarray analysis, and electrophysiologic studies. A flow diagram is presented in Figure 3 which proposes the sequential events at the time of GIP-34/GIP-8 contact with the cancer cell surface, followed by a cascade of signal transduced steps within the cytosol, resulting in the blockage of transnuclear passage of transcriptional or effector agents (not GIPs) into the nucleus.

Following the addition of GIP-34 into cancer cell culture dishes, the 34-mer attaches to the outer bilipid layer of the cell surface perturbing that interface and affecting fluidity of the lipid bilayer [28]. Studies by Eisele and MacColl *et al.* [13-16] and Mizejewski and Butterstein [36] demonstrated that GIP-34 can increase its alpha-helix content by 3-fold in the presence of 50% trifluoroethanol (TFE), a compound known to convert peptides into increased helical configurations, if they possess the innate propensity for such a secondary structure. Since TFE has been shown to produce a lipid-like micro-environment, alpha helical-converted GIP-34 forms could be predicted to have high cell membrane penetrance capabilities. Indeed, *in vivo* electrophysiological whole cell Sharp microelectrode and Patch-Clamp microelectrode measurements in tumor cells (MCF-7 breast cancer and LNCap prostate cancer) showed that GIP-34 acts both as a pore-forming/cell penetrating peptide and as a channel blocker (Figure 2) [28]. Thus, GIP-34 has both capabilities depending on its peptide molar concentration. The ability to increase a peptide's alpha-helix configuration permits it to corkscrew itself into the plasma membrane, thus disrupting and perturbing the integrity of the lipid bilayer cell membrane. Thus, it is highly likely that GIP-34 can undergo cell uptake by other means (*i.e.*, cell penetration) in addition to cell surface receptor binding. When cell penetration is compared to receptor binding, observations by Mizejewski and MacColl [19] and Cohen [45] reported that GIP-34 could gain cell entrance within 1–5 minutes, in contrast to receptor mediated endocytosis of peptides which requires 10 to 30 minutes for completion [19,56,64,65] (see below).

In order to study cell cycle events, GIP-34 was administered to MCF-7 cells for 8 days and subjected to a human global RNA microarray analysis of MCF-7 cancer cell lysates (Table 2) demonstrated that GIP-34 down-regulated the RNA of multiple cell cycle proteins [32,33]. As shown in Table 2, GIP-34 was found to down-regulate the RNA of a variety of kinase regulators of the cell cycle machinery including cyclin-E (4-fold reduction), SKP2 (4-fold reduction), Checkpoint Suppressor-1 (9-fold reduction), Establishment of Cohesion-1, (9-fold reduction), transcription Dp-1 (4-fold) and CDC20 (4-fold). All these cell cycle-related proteins are involved in G1 to S-phase arrest and to prevent further transition to mitotic cell division (Table 2). Secondly, GIP-34 was found to down-regulate the RNA of several ubiquitin E3 ligases which are involved in ubiquitin attachment and subsequent proteolytic degradation of p27 Kip and p21 Cip cell cycle inhibitors. One cause of Herceptin resistance has been attributed to the loss of p27; hence, GIP-34's down-regulation and suppression of p27 proteolysis would aid in overcoming Herceptin resistance [66,67]. Also, infusion of Herceptin into some patients can reportedly damage heart tissue and cause cardiac arrhythmias (arrest) due to Herceptin's continual activation of multiple KQT potassium channels in heart muscle [68,69]. In Table 3, it is shown that GIP-34 can down-regulate KQT potassium channels ((KCNQ3) III-3) by 4-fold as displayed in the RNA microarray analysis [70]. The ubiquitin-related proteolytic proteins' RNA down-regulated in the microarray analysis were F-Box/WD40-Domain-10 (15-fold reduction), SUMO/Sentrin/SMT3 (2-fold reduction), ubiquitin specific-49 (2-fold reduction), ubiquitin ligase protein complex (2-fold reduction), and Ring Finger Ligase-TRIM62 (3 fold reduction). Taken together, the above microarray data suggest that GIP-34 inhibits cancer cell growth by both S-Phase cell cycle arrest and prevention of cyclin inhibitor degradation by ubiquitinization and subsequent proteosomal processing. It can also be noted in Table 2, that GIP-34 down-regulated the RNA of several ion channels, including both potassium and calcium types.

It has been determined that a time exposure of GIP-34 treatment was critical in order to accomplish growth cessation of cancer cells. In these reports, a single treatment of GIP-34 proved to be insufficient over a period of 7 days [32,33,45]. For example, cancer cells were harvested and assayed at two days following a single treatment and growth arrest was clearly evident. However, five days later without further peptide treatment, the inhibitor effect was not observed. The above mentioned perturbance of the cell membrane, the increased electrochemical cell membrane potential and decreased resistance, together with the cell cycle S-Phase arrest and p27 proteolytic protection, was sufficient to suppress growth for at least the first two days, but was insufficient for five additional days in the absence of peptide. Thus, in cell culture studies, GIP-34 treatment of cancer cells should be maintained for at least eight days with alternate day treatment of the 34-mer peptide to achieve continued peptide exposure to the cells. Such a treatment regimen *in vivo* would lend itself to use of slow-release pellets or osmotic pumps for cancer therapy.

## 5. Proposed Mechanism of Action: GIP-8/AFPep

The mechanism of action of GIP-8 differs somewhat from that of GIP-34 regarding its inhibition of E-stimulated growth, but is similar in action to its basal growth inhibition of cancer cell growth. Similar to GIP-34, the 8-mer peptide when administered to MCF-7 cells, attaches to the outer bilipid layer of the plasma membrane and is thought to bind to or near a cell surface receptor in juxtaposition to a cation channel in a protein signaling cluster [71,72] (Figure 3). Electrophysiologic data has shown that GIP-8 in human glioblastoma cells blocked an outward voltage-activated K^+^ channel current [28] (Figure 3) demonstrating that GIP-8 is capable of acting as an ion channel blocker. In this regard, GIP-8 resembles insect venom peptides reported to be short peptides (8–12 AA) which are active at 10^−5^ to 10^−7^ M concentrations to form barrel-stave-like aggregated oligomers present at the cell surface membrane in areas (receptors) juxtaposed to ion channels [73]. The presence of such peptide aggregates distort the cell membrane bilayer at the area of impact, and cause membrane thinning by a redistribution of lateral stress components (polar head groups, acyl chains, cholesterol) within the bilayer extending to and blocking the ion channel. Since estradiol is known to activate and open K^+^ ion channels in the cell membrane, one part of the mechanism of action of GIP-34 and GIP-8 could result from K^+^ ion channel blockage [74]. Estradiol has been shown to open potassium channels leading to increased cell proliferation and growth of breast cancer cells [75]. The opening of voltage-gated K^+^ channels has indeed been correlated with growth and cell cycle progression in a multitude of human cancers including ovary, breast, bladder, neuroblastomas, and melanoma [76-84]. Furthermore, a direct association has been reported between cell membrane potential (depolarization) and human breast cancer growth inhibition studied using K^+^ ion channel blockers [81,82].

It has also been reported that the 8-mer peptide can inhibit the growth of MCF-7 cells stimulated by epidermal growth factor (EGF) as well as estradiol [60,61]. In that study, it was shown that GIP-8 interfered with enzymatic activities of the MAPK-kinases that were activated by the EGF/EGF-receptor interaction. Similar to estradiol, it is known that cells stimulated by EGF induce voltage-gated K^+^ ion channel opening in both normal and cancer cells, thus providing an underpinning of the mechanism of action of GIP-8 [74,85]. Once in the cytoplasm, GIP-8 has been demonstrated to inhibit the E-stimulated phosphorylation of serine-118 of human ER in lysates of T47D breast cancer cells; it also interferes with the phosphorylation of p53, an event that inactivates this tumor suppressor [56]. Further data provided by Sierralta *et al.* showed that GIP-8 caused an increase in cytoplasmic P21 Cip levels, but had no effect on E-cadherin expression, apoptosis, and endogenous aromatase activity in both MCF-7 and ZR75-1 human breast cancer cells [63]. The authors of this latter report stated that GIP-8 suppressed cell cycle progression at the S to G2 phase via regulation of the p21Cip cyclin inhibitor. Taken together, these data imply that the mechanism of action of GIP-8 in the growth suppression of E-stimulated breast cancer is the result of: (1) blockage of voltage-gated K^+^ ion channels; (2) interference of EGFR- and E2-stimulated MAPK-kinase activities; (3) inhibition of serine-118 human ER phosphorylation; (4) interference of P53 tumor suppressor phosphorylation and (5) increase in p21 Cip levels that impede cell cycle stage progression.

In contrast to the E-stimulated breast cancer cell growth, the inhibition of basal cancer cell growth by GIP-8 does not appear to require the presence of the human estrogen receptor and its stimulatory co-activators. Once GIP-8 binds to the cell surface and enacts K^+^ ion channel blockage, it appears to disrupt the signal transduction cascades that result in the cessation of cell cycle progression and subsequent mitosis (Figure 3). The blockage of K^+^ ion channels and the subsequent cell membrane depolarization known to impede cancer cell proliferation are consistent with these data [80-83]. Although the mechanism of cancer cell growth impairment might involve cell cycle RNA down-regulation, an RNA microarray analysis using GIP-8 has not yet been performed. Certainly, GIP-8 fulfills the criteria as a growth inhibitory agent that could enhance p21 Cip binding to Cdk2 complexes, block degradation of the p21 cell cycle inhibitor, and prevent the migration of Cdk2 kinase entrance into the nucleus. Following GIP-8 treatment, the p21-Cdk2 complex could remain cytosolic-bound and be incapable of promoting cell cycle transition of S to G2 phase, resulting in arrest of the cell cycle.

## 6. Current Developments and Future Prospects

The GIP-34 and its peptide synthetic fragments were designed and derived from a naturally-occurring fetal protein (AFP) present during human pregnancy. The encrypted GIP segment lies buried within the protein molecule until AFP encounters stress/shock environments; GIP is then exposed in the conformationally transformed fetal protein (Figure 1). The GIP segment functions to prevent and suppress inappropriate fetal growth until proper signaling transduction cascades can be re-established in the developing embryo/fetus. Such GIP surveillance might account for the low prevalence of embryonic/fetal defects and tumors during pregnancy. Due to the low nanogram levels (6 ng/mL) present in circulating adult HAFP, transformed AFP and its exposed GIP segments are not available to adult humans to aid in bodily defense from cancer and benign growths. Thus, the initial goal of the GIP technology platform was to eventually make GIP-34 and its growth inhibitory subcomponents available as peptide drugs for adults afflicted with benign hyperplasia and cancer growth as seen in hepatic, reproductive, and gastrointestinal tumors. Currently, GIP and its derived segments are under development for conjugation to chemotherapeutic drugs by means of covalent bonding to side chain residues. Preliminary *in vitro* studies using GIP-8 have already shown success in the conjugation of GIP to doxorubicin and GIP-34 has been chemically linked to radioiodine and technicium-99 for *in vivo* use in clinical imaging of mammary tumors [45]. Since GIP-34 binds iron and other heavy metals (copper, zinc, cobalt), it has been utilized in preliminary studies in clinical imaging procedures (MRI, CAT, *etc.*). Because GIP binds to Congo Red and anilinonapthalene-sulfonic acid (ANS), it could be considered as an agent for organ function tests [18]. Finally, the development of GIP-34 as a DNA-PK inhibitor peptidomimetic is in its beginning stages.

The hurdles encountered in the commercial developing and marketing of GIP technology products are largely involved with the cost of synthesis and manufacturing methods for peptides *versus* small molecule drugs. Although research peptides are highly regarded as agents for revealing molecular and cellular targets, large pharmaceutical companies have been slow to embrace peptide-based drugs, possibly due to their short half-lives via proteolytic degradation. Nonetheless, biotech companies and pharmaceutical firms are gradually incorporating peptides into their pipelines and providing them to the medical community. At the present time, GIP-34 and GIP-8 are being subjected to computer (silica-based) modeling of peptide-to-protein docking software from biotech companies (*i.e.*, Serometrix) for the discovery of new molecular, cell surface, and subcellular targets. Regarding clinical trials, GIP-34 is undergoing pre-clinical trials and has progressed to large animal studies involving dogs and cats. Finally, GIP-8 has been admitted into the application stages for Phase-I human breast cancer clinical trials at a large eastern United States university medical center.

## 7. Conclusions

From the preceding discussion, it is evident that GIP-34 and GIP-8 can inhibit cancer cell growth by different pathways, but produce similar outcomes. While each peptide may enter the tumor cell by slightly different routes, the endpoints of cell cycle arrest, and prevention of cyclin inhibitor degradation are similar. Since GIP-34 contains the GIP-8 fragment within its AA sequence, the use of GIP-34 would seem more efficient and could utilize both pathways; however, the cost of synthesis of GIP-34 is much higher than GIP-8. Both the linear and cyclic configurations of both peptides exhibit long shelf-life and the lyophilized powder can be stored in a dry state, at room temperature, in the dark for long periods of time. GIP-8, and possibly GIP-34, can be given by oral administration and could be developed into a pill-form for human medication. Both peptides are well-tolerated in animal studies, are mechanistically novel, and can be used in combination with or conjugated to chemotherapeutic drugs such as tamoxifen and doxorubicin.

A major advantage of using both peptides is that no toxic side-effects have ever been observed or reported in over 1,000 animals utilized in pre-clinical trials, even at extremely high doses [22,34,36]. Some of this effect can be explained by the cytostatic rather than cytotoxic activity of the GIPs. The evidence for the lack of toxicity in animals was determined by observation and measurement of body weights, cage activity, fur texture, individual organ weights and histology, behavioral activities, longevity, and death recordings [24,54,55]. Both peptides can complement the use of tamoxifen by alleviating the uterine hyperplasic side effects when given in peptide-tamoxifen combinations [21,36]. Tamoxifen binds to the human ER but does not activate it, while GIP-8 is able to inhibit the serine-118 phosphorylation of the ER [21,49,54]. In comparison GIP-34, like tamoxifen, is capable of binding to the ER [11].

The 34-mer peptide has the advantage of being both a cell penetrating peptide (CPP) and a channel blocker depending on peptide concentration as demonstrated by electrophysiologic studies. The CPPs are known to gain entrance into cancer cells by disrupting or disturbing the bilipid cell surface and cork-screwing itself into the plasma membranes of cells displaying an overall net negative cell surface charge as see in many cell cancer types [86,87]. Hence, cells destined for apoptosis, including cancer cells, are known to undergo a cell membrane “lipid inversion” by switching sphingomyelin and/or phosphatidylcholine for phosphatidylserine, thus shifting a negative charge to the cancer cell apical surface [88]. The negative-charged cell surface not only flags cells for targeted apoptosis, but also designates the cell as a candidate for cell penetration, and transmembrane passage. Thus, CPPs do not attach or bind to positively charged normal cells, but rather to cells displaying a higher net negative charge on their surface, as found in cancer cells [45]. This phenomenon could provide a basis of target specificity for targeting cancer cells, but not bystander cells. The ion channels affected by CPPs are largely voltage-dependent and are selective for cations such as Ca^++^, K^+^, and Na^+^ ions. GIP-34 has been confirmed to affect voltage-gated K^+^-channels as shown in the RNA microarray data (Table 2) and in the electrophysiology studies (Figure 2). In contrast, short amino acid sequence peptides like GIP-8 do not show CPP activities, but instead exhibit channel blocker activity that could eventually result in down-regulation of ion channels.

Another advantage in the use of GIP-34 stems from its potential use as radiosensitizer and chemosensitizer agents as demonstrated in previous publications. One such report involved irradiated thymocytes described by Mizejewski *et al.* [34,36]. The results showed that gamma X-ray exposure of mouse thymocytes incubated overnight in the presence of 10^−8^ to 10^−10^ M GIP-34 enhanced apoptosis in the irradiated thymocytes. These results suggested that GIP-34 might be utilized as a tumor cell radiosensitizing agent prior to or during chemotherapy. In other studies, both GIP-34 and GIP-8 were employed as chemosensitizing agents when used prior to or in combination with tamoxifen or doxorubicin [28,45]. In both instances, the anti-cancer effect of GIP combined or conjugated to the chemotherapy drug was enhanced in T47D breast cancer and glioblastoma cells; furthermore, it may be possible that drug resistance to chemoagents could be bypassed. Finally, both peptides might serve as allosteric drugs in that the peptides can dock to target protein molecules at a site other than that of the major ligand binding pocket as demonstrated by computer modeling of peptide (GIP)-to-protein docking [89-91].

An unexpected advantage of using both GIP fragments was found to be enhancement of the immune response to lectins such as Con-A, and to serve as AFP antigenic epitopes for T-cell sensitization of cell-mediated immune responses. In the induction of a T-cell mediated immune response, two juxtaposed sequences on GIP-34 were demonstrated to serve as epitopes for antigen presentation of dendritic cells to T-cells to induce cytotoxic lymphocytes directed against AFP bound to hepatoma cells [37-39]. Thereby, GIP-34 should be growth suppressive against hepatoma cells in culture and a recent report confirmed this prediction [45]. In other reports, both GIP segments were found effective as anti-angiogenic factors during chick development and in mouse cancer cell cultures [17,36]. Furthermore, GIP-34 and GIP-8 may prove to be efficacious as breast cancer therapeutic agents if used in conjunction with infused tamoxifen therapy because of their anti-uterotrophic (hyperplasia) properties; additionally, GIP-34 could be used as an inhibitor of platelet aggregation to aid in preventing blood clots observed in human patients undergoing tamoxifen treatment [36]. Finally, GIP-34 could be effective as an anti-metastatic agent due to its ability to inhibit cell spreading, platelet aggregation, and cellular adhesion to ECM proteins [34].

## Figures and Tables

**Table 1.** Historical background and development of the human alpha-fetoprotein (AFP) derived growth inhibitory peptides 34-mer (GIP-34) and 8-mer (GIP-8); relevance to biological activities (Abbreviations: FMOC = peptide synthesis method; MCF-7, T47D, MBA-MB-231 = human breast cancer cell lines; SKOVI = human ovarian cancer cell line; NYLAR = New York State mouse cell line; GWI-1 = New York State mouse mammary tumor; E2 = estradiol; C3H = inbred mouse cell line).

**Year**	**Organism, Cell Culture or Chemical**	**Cell, Tissue, or Synthesis**	**Biological or Biochemical Activity/Role of GIP-34**	**Author and References**
1990– 1993	Mouse, human, peptide sequence	Immature uterus, GIP-34 synthesis, MCF-7 breast cancer cells	Discovery of GIP-34; suppression of estrogen-stimulated uterine and cancer growth	Mizejewski *et al.* [12,99,100]
1994– 1995	Human AFP-derived peptides GIP-34 and GIP-8	FMOC GIP-34 peptide synthesis, immature mouse uterus; FMOC-synthesized GIP-8, MCF-7 cells	Trypsin digest of GIP-34; GIP-8 and two subfragments were produced and bioassayed	Mizejewski *et al.* [12,92,94]
1996– 1997	Mouse, Human AFP AFP-derived peptides, GIP-34, GIP-8	Immature uterus, MCF-7 breast cancer cells, NYLAR mouse ascites tumor-6WI-1	-suppression of estrogen-stimulated uterine growth; two U.S. Patents-suppression of ascites breast tumor growth; -suppression of breast cancer cell proliferation	Mizejewski *et al.* [3,12,101]
1998	Human AFP-derived peptides, transformed serum HAFP	AFP-derived GIP-34, GIP-8 and fragments	U.S. patent issued for GIP-34, GIP-8, GIP-12, and GIP-14	Mizejewski, Richardson *et al.* [95,96,102]
1999	Human AFP-derived peptides, mouse ascites fluid, MCF-7 cell focus assay	AFP-derived GIP-34, mouse mammary tumor; linear GIP-8	GIP-34 peptides bind to Human Estrogen Receptor; GIP-34, GIP-8-suppresses breast tumor growth	Vakharia [11]; Butterstein *et al.* [93]; Mizejewski *et al.* [47,97]
2000	Human AFP-derived peptide, mouse ascites; GIP-12, GIP-14, GIP-8	AFP-derived GIP-34; GIP-8 synthesized in a cyclic form	Peptides GIP-34 and GIP-12 binds to human estrogen receptor, GIP-14 binds estradiol and suppresses breast cancer growth	Vakharia and, Mizejewski [11]; Mesfin *et al.* [23]
2001	Human AFP-derived peptide, immature mouse	AFP-derived GIP-34 and GIP-8, mouse uterus, mammary ascites fluid and tumor cells	-study of GIP-34 tumor, dimer/trimer oligomer activities GIP-8-suppresses uterine growth stimulated by E2	Mizejewski [2]; Eisele *et al.*, MacColl *et al.* [13-15]; Mesfin *et al.* [48]
2002	Human cancer cell cultures, GIP-34 and GIP-8 amino acid analogs	Prostate cancer, breast cancer cells, MCF-7, T47D, MBA-MB-231	Peptide GIP-34 suppresses growth of prostate and GIP-8 suppresses breast cancer cells	Mizejewski [5]; Caceres *et al.* [20]; Bennett *et al.* [21]; Mesfin *et al.* [48]
2003	AFP-derived peptide (GIP-34), immature rat, human breast cancer xenografts, chicken, mouse pups, and pregnant linear Dams, GIP-8 fragments	Human derived peptide, estrogen stimulated uterus, MCF-7, 6WI-1 Breast cancer cells, Chick embryo, pups and pregnant mice—GIP-34. GIP-8 and E2-toxicity and insulin birth defects	Peptide binds cobalt and zinc; suppresses E2-supported uterine growth and basal growth of ductal and glandular breast cancer cells; GIP peptide suppresses insulin-induced birth defects; inhibits estrogen-induced uterine growth and fetotoxicity	Butterstein *et al.* [16-17], Mizejewski *et al.* [19]; Andersen *et al.* [55]
2004	GIP-34, GIP-8 human cell cancer cultures and erythrocytes, mouse pups, binding and polymer assays	HELA cells, immature mouse uterus, MCF-7 breast cancer cells, GIP-34, GIP-8	Peptide GIP-8 optimal dose for growth suppression; pharmacophore discovered GIP-34 binding of tubulin, Congo Red and ANS fluorescent markers	Mizejewski *et al.* [4,18] DeFreest *et al.* [49]
2005	Mouse mammary tumors, various human tumors, ECM protein adhesion assays, GIP-34, GIP-8	GWI-1 acsites mouse mammary of tumors, MCF-7 breast cancer cell, colon, ovary, prostate, melanoma, *etc.*; Nitrosouria-induced rat breast cancer suppression by GIP-8	Peptide GIP-34 suppresses growth of 38 of 60 human tumors, mouse ascites tumors, and adhesion of cancer cells to ECM, cancer cell migration invasion, and metastasis; GIP-8 is orally active; cyclin E1 inhibited	Bennett *et al.* [24]; Mizejewski *et* al. [25,35,36]; Turk *et al.* [32]; Muehlemann *et al.* [34]
2006	Chick embryo, frog tadpole, artemia brine shrimp; cancer mouse organs; human cancer, Ache enzymes, cytochromes, and enzymes	Liver, adult and immature uterus, breast cancer cells, human platelets, MCF-7, GI-101 Breast tumors	GIP-34 peptide inhibits cell migration, spreading, adhesion, growth processes of lower vertebrates, cell shape changes (cytoskeleton), and contact inhibition. GIP-8, found as orally active	Bennett *et al.* [24]; Mizejewski *et al.* [25]
2007	Human GIP-34 and GIP-8 peptides, and AFP receptor; mouse and human cancer cell cultures, tamoxifen treated cells	MCF-7, T-47D, MDA-MB-231, MDA-MB-435, breast cancers, PC-3 prostate; liver, glial tumors, uterine hyperplasia suppressed by GIP-8	Peptides GIP-34 and GIP-8 suppresses growth of various cancers; inhibits angiogenesis and ECM protein binding; GIP-8 does not bind to the AFP receptor	Mizejewski [22]; Kirschner *et al.* [26]; Andersen *et al.* [58]
2008	Human AFP full-length protein and cyclized peptides GIP-34 and GIP-8	AFP antigenic epitopes, discovered; MCF-7, ZR75-1, MCB-MB-231 breast cancer cells	Peptides GIP-14 and GIP-8 induce cell-mediated response and cytokine secretion. GIP-8 suppression	Butterfield *et al.* [40-42]; Torres *et al.* [60]; Sierralta *et al.* [61,63]
2009	Human full-length AFP derived peptides; Atlas for AFP-derived peptides	GIP-34 peptide and other peptides, cyclized GIP-8, beta Hairpin turn as active binding site	Functional mapping of GIP-34, GIP-12, GIP-14, and GIP-8, breast cancer xenografts	Mizejewski *et al.* [1,6]; Shields [27]; Butterfield *et al.* [41], Joseph *et al.* [50]; Tower *et al.* [59]; Torres *et al.* [62]
2010	Human cancer cell lines, tumor-bearing C3H mice, chemo-drug conjugation; Human cancer cell culture lines	GIP-34 and GIP-8 peptides, MCF-7, ovarian SKOVI, doxorubicin; Follicular thyroid carcinoma, T47D; AFP peptides were proposed for Human therapeutic use	Peptide targeted delivery to various cancer cells *in vivo* and *in vitro*; radiolabeled GIP-34 biodistribution; Peptide (GIP-34) suppresses thyroid cancer cell migration, invasion, and metastasis	Mizejewski [10]; Mizejewski *et al.* [28,45]; Hua *et al.* [98]

**Table 2.** Global RNA microarray data: Transcripts displaying 1.0 or larger log fold (log base 2.0) decrease for genes associated with cell division and proliferation processes, ubiquitization, and cation channels obtained from Human MCF-7 breast cancer cells *in vitro* *.

**Gene Title**	**Fold Decrease (−)**	**Cell Function**
**I. Cell Cycle Regulation**		
1. Calpain (LOC 441200)	−32.5	Cell cycle progression
2. F-Box/Wd40, Domain-10 (FBXW10)	−14.9	P27 degradation pathway
3. Serine/Threonine Kinase-33 (STK33)	−9.2	SH3 protein kinase
4. Establishment of Cohesion-1, Homolog (ESC02)	−9.2	DNA replication
5. Checkpoint Suppressor-1 (CHES1) (FOXN3)	−9.2	S-phase checkpoint
6. Cyclin-E **	−4.6	Regulates G-S transition
7. SKP2 **	−4.3	Mediates p27 degradation
8. Transcription Dp-1 (TFDP1)	−4.3	Binds E2F-1; G1 to S
9. CDC20 Cell Division Homolog	−4.3	Activates ubiquitins
10. Triple Function Domain (TRIO)	−3.7	Actin remodeling
11. Histone-1, H4g (HIST1H4G)	−3.2	DNA repair/replication
12. Fanconi Anemia-D2 (FRANCD2)	−2.0	DNA repair/synthesis
**II. Ubiquitin-associated Proteases**		
1. Triparite Motif-containing-62 (TRIM62)	−3.0	Fing finger ligase
2. SH3 Domain Protein (EVE1)	−2.3	ADAMS regulation
3. Samd and SH3 containing Domain-1 (SASH1)	−2.1	Breast tumorigenesis
4. SUMO1/Sentrin/SMT3 Specific Protease (SENP3)	−2.1	Lysine targeting ubiquitin
5. Ubiquitin Specific Protease-49 (MGC20741)	−2.1	Ubiquitin enzyme
6. Ubiquitin Ligase Protein Comples (KIAA0804)	−2.1	Protein degradation
**III. Channel Associated Proteins**		
1. Potassium Voltage-gated Channel (KCNB2)	−8.0	Shab ion channel
2. Transmembrane Channel Like 5 (TMC5)	−5.2	Ion transporter
3. Potassium Voltage-gated Channel, KQT-like (KCNQ3)	−4.0	Cation signaling
4. Calcium Channel, Voltage dependent of 2 (CACNA2D4)	−2.0	Calcium signaling
5. Calcium/Calmodulin-dependent Kinase (CAMK2B)	−1.9	Calcium regulation
6. Calcineurin A gamma (PPP3CC)	−1.8	Calcium phosphatase 3 protein
7. Calcium Channel, Voltage Dependent (CACNC6)	−1.8	Calcium transport

* Expression of 716 transcripts was significantly altered in MCF-7 cells after 8 days of treatment with GIP as compared to treatment with the scrambled peptide. Four hundred thirty RNAs were down regulated, while 286 RNAs were up regulated;

** = real time PCR. Data provided by Kathleen Arcaro, University of Massachusetts, Amherst, MA [32,33].

## References

[1] Mizejewski G. (2009). Mapping of structure-function peptide sites on the human alpha-fetoprotein amino acid sequence. Atlas Genet. Cytogenet. Oncol. Haematol..

[2] Mizejewski G.J. (2001). Alpha-fetoprotein structure and function: Relevance to isoforms, epitopes, and conformational variants. Exp. Biol. Med. (Maywood)..

[3] Mizejewski G.J. (1997). Alpha-fetoprotein as a biologic response modifier: Relevance to domain and subdomain structure. Proc. Soc. Exp. Biol. Med..

[4] Mizejewski G.J. (2004). Biological roles of alpha-fetoprotein during pregnancy and perinatal development. Exp. Biol. Med. (Maywood)..

[5] Mizejewski G.J. (2002). Biological role of alpha-fetoprotein in cancer: Prospects for anticancer therapy. Expert Rev. Anticancer Ther..

[6] Mizejewski G.J. (2009). Alpha-fetoprotein (AFP)-derived peptides as epitopes for hepatoma immunotherapy: A commentary. Cancer Immunol. Immunother..

[7] Mizejewski G.J. (2007). Physiology of alpha-fetoprotein as a biomarker for perinatal distress: Relevance to adverse pregnancy outcome. Exp. Biol. Med. (Maywood)..

[8] Li M., Li H., Li C., Guo L., Liu H., Zhou S., Liu X., Chen Z., Shi S., Wei J. (2009). Cytoplasmic alpha-fetoprotein functions as a co-repressor in RA-RAR signaling to promote the growth of human hepatoma Bel 7402 cells. Cancer Lett..

[9] Li M., Li H., Li C., Zhou S., Guo L., Liu H., Jiang W., Liu X., Li P., McNutt M.A. (2009). Alpha fetoprotein is a novel protein-binding partner for caspase-3 and blocks the apoptotic signaling pathway in human hepatoma cells. Int. J. Cancer.

[10] Mizejewski G.J. (2011). Therapeutic use of human alpha-fetoprotein in clinical patients: Is a cancer risk involved?. Int. J. Cancer.

[11] Vakharia D., Mizejewski G.J. (2000). Human alpha-fetoprotein peptides bind estrogen receptor and estradiol, and suppress breast cancer. Breast Cancer Res. Treat..

[12] Mizejewski G.J., Dias J.A., Hauer C.R., Henrikson K.P., Gierthy J. (1996). Alpha-fetoprotein synthetic peptides: Characterization and assay of an estrogen-sensitive growth regulatory segment. Mol. Cell. Endocrinol..

[13] Eisele L.E., Mesfin F.B., Bennett J.A., Andersen T.T., Jacobson H.I., Vakharia D.D., MacColl R., Mizejewski G.J. (2001). Studies on analogs of a peptide derived from alpha-fetoprotein having antigrowth properties. J. Pept. Res..

[14] Eisele L.E., Mesfin F.B., Bennett J.A., Andersen T.T., Jacobson H.I., Soldwedel H., MacColl R., Mizejewski G.J. (2001). Studies on a growth-inhibitory peptide derived from alpha-fetoprotein and some analogs. J. Pept. Res..

[15] MacColl R., Eisele L.E., Stack R.F., Hauer C., Vakharia D.D., Benno A., Kelly W.C., Mizejewski G.J. (2001). Interrelationships among biological activity, disulfide bonds, secondary structure, and metal ion binding for a chemically synthesized 34-amino-acid peptide derived from alpha-fetoprotein. Biochim. Biophys. Acta.

[16] Butterstein G., MacColl R., Mizejewski G.J., Eisele L.E., Meservey M. (2003). Biophysical studies and anti-growth activities of a peptide, a certain analog and a fragment peptide derived from alpha-fetoprotein. J. Pept. Res..

[17] Butterstein G., Morrison J., Mizejewski G.J. (2003). Effect of alpha-fetoprotein and derived peptides on insulin- and estrogen-induced fetotoxicity. Fetal Diagn. Ther..

[18] Mizejewski G., Smith G., Butterstein G. (2004). Review and proposed action of alpha-fetoprotein growth inhibitory peptides as estrogen and cytoskeletal-associated factors. Cell Biol. Int..

[19] Mizejewski G.J., MacColl R. (2003). Alpha-fetoprotein growth inhibitory peptides: Potential leads for cancer therapeutics. Mol. Cancer Ther..

[20] Caceres G., Dauphinee M.J., Eisele L.E., MacColl R., Mizejewski G.J. (2002). Anti-prostate cancer and anti-breast cancer activities of two peptides derived from alpha-fetoprotein. Anticancer Res..

[21] Bennett J.A., Mesfin F.B., Andersen T.T., Gierthy J.F., Jacobson H.I. (2002). A peptide derived from alpha-fetoprotein prevents the growth of estrogen-dependent human breast cancers sensitive and resistant to tamoxifen. Proc. Natl. Acad. Sci. USA.

[22] Mizejewski G.J. (2007). The alpha-fetoprotein-derived growth inhibitory peptide 8-mer fragment: Review of a novel anticancer agent. Cancer Biother. Radiopharm..

[23] Mesfin F.B., Bennett J.A., Jacobson H.I., Zhu S., Andersen T.T. (2000). Alpha-fetoprotein-derived antiestrotrophic octapeptide. Biochim. Biophys. Acta.

[24] Bennett J.A., DeFreest L., Anaka I., Saadati H., Balulad S., Jacobson H.I., Andersen T.T. (2006). AFPep: An anti-breast cancer peptide that is orally active. Breast Cancer Res. Treat..

[25] Mizejewski G.J., Eisele L., Maccoll R. (2006). Anticancer *versus* antigrowth activities of three analogs of the growth-inhibitory peptide: Relevance to physicochemical properties. Anticancer Res..

[26] Kirschner K.N., Lexa K.W., Salisburg A.M., Alser K.A., Joseph L., Andersen T.T., Bennett J.A., Jacobson H.I., Shields G.C. (2007). Computational design and experimental discovery of an antiestrogenic peptide derived from alpha-fetoprotein. J. Am. Chem. Soc..

[27] Shields G.C. (2009). Computational approaches for the design of peptides with anti-breast cancer properties. Future Med. Chem..

[28] Mizejewski G.J., Garnuszek P., Maurin M., Mirowski M., Cohen B.D., Pioiecz B.D., Polypanova G.A., Makaros V.A., Severin E.S., Severin S.E. Cancer cell targeted delivery of growth inhibitory peptides derived from human alpha-fetoprotein: Review of an international multi-center collaborative study.

[29] Tamba Y., Ariyama H., Levadny V., Yamazaki M. (2010). Kinetic pathway of antimicrobial peptide magainin 2-induced pore formation in lipid membranes. J. Phys. Chem. B.

[30] Sabourin J., Cognard C., Constantin B. (2009). Regulation by scaffolding proteins of canonical transient receptor potential channels in striated muscle. J. Muscle Res. Cell Motil..

[31] Strobl J.S., Wonderlin W.F., Flynn D.C. (1995). Mitogenic signal transduction in human breast cancer cells. Gen. Pharmacol..

[32] Turk C., Wong C., Gozgit J.M., Muehlemann M., Reece M.T., Mizejewski G.J., Arcaro K.F. (2006). Alpha-fetoprotein derived growth inhibitory peptide (GIP) inhibits expression of cyclin E1. Proc. Am. Assoc. Cancer Res..

[33] Turk C., Wong C.M., Gozgit J.M., Fagen-Solis K., Mizejewski G.J., Arcaro K.F. Alpha-fetoprotein-derived peptide decreases cyclin-E expression and P27 (KIP1) degradation in MCF-7 breast cancer cells.

[34] Muehlemann M., Miller K.D., Dauphinee M., Mizejewski G.J. (2005). Review of Growth Inhibitory Peptide as a biotherapeutic agent for tumor growth, adhesion, and metastasis. Cancer Metastasis Rev..

[35] Mizejewski G.J., Muehlemann M., Dauphinee M. (2006). Update of alpha fetoprotein growth-inhibitory peptides as biotherapeutic agents for tumor growth and metastasis. Chemotherapy.

[36] Mizejewski G.J., Butterstein G. (2006). Survey of functional activities of alpha-fetoprotein derived growth inhibitory peptides: Review and prospects. Curr. Protein Pept. Sci..

[37] Butterfield L.H., Meng W.S., Koh A., Vollmer C.M., Ribas A., Dissette V.B., Faull K., Glaspy J.A., McBride W.H., Economou J.S. (2001). T cell responses to HLA-A*0201-restricted peptides derived from human alpha fetoprotein. J. Immunol..

[38] Butterfield L.H., Koh A., Meng W., Vollmer C.M., Ribas A., Dissette V., Lee E., Glaspy J.A., McBride W.H., Economou J.S. (1999). Generation of human T-cell responses to an HLA-A2.1-restricted peptide epitope derived from alpha-fetoprotein. Cancer Res..

[39] Meng W.S., Butterfield L.H., Ribas A., Heller J.B., Dissette V.B., Glaspy J.A., McBride W.H., Economou J.S. (2000). Fine specificity analysis of an HLA-A2.1-restricted immunodominant T cell epitope derived from human alpha-fetoprotein. Mol. Immunol..

[40] Butterfield L.H., Ribas A., Dissette V.B., Lee Y., Yang J.Q., De la Rocha P., Duran S.D., Hernandez J., Seja E., Potter D.M. (2006). A phase I/II trial testing immunization of hepatocellular carcinoma patients with dendritic cells pulsed with four alpha-fetoprotein peptides. Clin. Cancer Res..

[41] Butterfield L.H., Ribas A., Meng W.S., Dissette V.B., Amarnani S., Vu H.T., Seja E., Todd K., Glaspy J.A., McBride W.H. (2003). T cell responses to HLA-A*0201 immunodominant peptides derived from alpha-fetoprotein in patients with hepatocellular cancer. Clin. Cancer Res..

[42] Butterfield L.H., Ribas A., Potter D.M., Economou J.S. (2007). Spontaneous and vaccine induced AFP-specific T cell phenotypes in subjects with AFP-positive hepatocellular cancer. Cancer Immunol. Immunother..

[43] Vollmer C.M., Eilber F.C., Butterfield L.H., Ribas A., Dissette V.B., Koh A., Montejo L.D., Lee M.C., Andrews K.J., McBride W.H. (1999). Alpha-fetoprotein-specific genetic immunotherapy for hepatocellular carcinoma. Cancer Res..

[44] Butterfield L.H. (2004). Immunotherapeutic strategies for hepatocellular carcinoma. Gastroenterology.

[45] Mizejewski G.J., Mirowski M., Garnuszek P., Maurin M., Cohen B.D., Poiesz B.J., Posypanova G.A., Makarov V.A., Severin E.S., Severin S.E. (2010). Targeted delivery of anti-cancer growth inhibitory peptides derived from human alpha-fetoprotein: Review of an International Multi-Center Collaborative Study. J. Drug Target..

[46] Mizejewski G.J. (2011). Review of the putative cell-surface receptors for alpha-fetoprotein: Identification of a candidate receptor protein family. Tumour Biol..

[47] Mizejewski G., Vakharia D., Richardson B.E. Binding of a human alpha-fetoprotein fragment to the estrogen receptor.

[48] Mesfin F.B., Andersen T.T., Jacobson H.I., Zhu S., Bennett J.A. (2001). Development of a synthetic cyclized peptide derived from alpha-fetoprotein that prevents the growth of human breast cancer. J. Pept. Res..

[49] DeFreest L.A., Mesfin F.B., Joseph L., McLeod D.J., Stallmer A., Reddy S., Balulad S.S., Jacobson H.I., Andersen T.T., Bennett J.A. (2004). Synthetic peptide derived from alpha-fetoprotein inhibits growth of human breast cancer: Investigation of the pharmacophore and synthesis optimization. J. Pept. Res..

[50] Joseph L., Bennett JA, Kirschner KN, Shields GC, Hughes J., Lostritto N, Jacobson HI, Andersen T. (2009). Antiestrogenic and anticancer activities of peptides derived from the active site of alpha-fetoprotein. J. Pept. Sci..

[51] Zhu J., Jiang J., Zhou W., Zhu K., Chen X. (1999). Differential regulation of cellular target genes by p53 devoid of the PXXP motifs with impaired apoptotic activity. Oncogene.

[52] Baptiste N., Friedlander P., Chen X., Prives C. (2002). The proline-rich domain of p53 is required for cooperation with anti-neoplastic agents to promote apoptosis of tumor cells. Oncogene.

[53] Li H.S., Montell C. (2000). TRP and the PDZ protein, INAD, form the core complex required for retention of the signalplex in Drosophila photoreceptor cells. J. Cell Biol..

[54] Parikh R.R., Gildener-Leapman N., Narendran A., Lin H.Y., Lemanski N., Bennett J.A., Jacobson H.I., Andersen T.T. (2005). Prevention of *N*-methyl-*N*-nitrosourea-induced breast cancer by alpha-fetoprotein (AFP)-derived peptide, a peptide derived from the active site of AFP. Clin. Cancer Res..

[55] Andersen T., Parikh R.R., Lemanski N., McLeod D., Georgekutty J., Saadati H., Fake J., Jacobson H.I., Bennett J.A. (2003). Safe and effective prevention of breast cancer by AFPep peptide analog of alpha-fetoprotein. Cancer Epidemiol. Biomarker. Prev..

[56] Pise-Masison C.A., Radonovich M., Sakaguchi K., Appella E., Brady J.N. (1998). Phosphorylation of p53: A novel pathway for p53 inactivation in human T-cell lymphotropic virus type 1-transformed cells. J. Virol..

[57] Kohlhaas S.L., Craxton A., Sun X.M., Pinkoski M.J., Cohen G.M. (2007). Receptor-mediated endocytosis is not required for tumor necrosis factor-related apoptosis-inducing ligand (TRAIL)-induced apoptosis. J. Biol. Chem..

[58] Andersen T.T., Georgekutty J., DeFreest L.A., Amaratunga G., Narendran A., Lemanski N., Jacobson H.I., Bennett J.A. (2007). An alpha-fetoprotein-derived peptide reduces the uterine hyperplasia and increases the antitumour effect of tamoxifen. Br. J. Cancer.

[59] Tower A.M., Trinward A., Lee K., Joseph L., Jacobson H.I., Bennett J.A., Andersen T.T. (2009). AFPep, a novel drug for the prevention and treatment of breast cancer, does not disrupt the estrous cycle or fertility in rats. Oncol. Rep..

[60] Torres C., Antileo E., Epunan M.J., Pino A.M., Valladares L.E., Sierralta W.D. (2008). A cyclic peptide derived from alpha-fetoprotein inhibits the proliferative effects of the epidermal growth factor and estradiol in MCF7 cells. Oncol. Rep..

[61] Sierralta W.D., Epunan M.J., Reyes J.M., Valladares L.E., Andersen T.T., Bennett J.A., Jacobson H.I., Pino A.M. (2008). A peptide derived from alpha-fetoprotein inhibits the proliferation induced by estradiol in mammary tumor cells in culture. Oncol. Rep..

[62] Torres C.G., Pino A.M., Sierralta W.D. (2009). A cyclized peptide derived from alpha fetoprotein inhibits the proliferation of ER-positive canine mammary cancer cells. Oncol Rep..

[63] Sierralta W.D., Epunan M.J., Reyes J.M., Valladares L.E., Pino A.M. (2008). A synthetic peptide derived from alpha-fetoprotein inhibits the estradiol-induced proliferation of mammary tumor cells in culture through the modulation of p21. Adv. Exp. Med. Biol..

[64] Lennon J., Bennett J., Andersen T., Barroso M. The anti-oncogenic activity of peptide AFPep is dependent on its mechanism of entry into breast cancer cells.

[65] Yu A., Malek T.R. (2001). The proteasome regulates receptor-mediated endocytosis of interleukin-2. J. Biol. Chem..

[66] Xu D., Wang L., Dai W., Lu L. (1999). A requirement for K^+^-channel activity in growth factor-mediated extracellular signal-regulated kinase activation in human myeloblastic leukemia ML-1 cells. Blood.

[67] Mukohara T. (2011). Mechanisms of resistance to anti-human epidermal growth factor receptor 2 agents in breast cancer. Cancer Sci..

[68] Nahta R., Takahashi T., Ueno N.T., Hung M.c., Esteva F.J. (2004). P27(kip1) down-regulation is associated with trastuzumab resistance in breast cancer cells. Cancer Res..

[69] Wickenden A.D., Zou A., Wagoner P.K., Jegla T. (2001). Characterization of KCNQ5/Q3 potassium channels expressed in mammalian cells. Br. J. Pharmacol..

[70] Seidman A., Hudis C., Pierri M.K., Shak S., Paton V., Ashby M., Murphy M., Stewart S.J., Keefe D. (2002). Cardiac dysfunction in the trastuzumab clinical trials experience. J. Clin. Oncol..

[71] Neve K.A. (2005). Double feature at the signalplex. Mol. Pharmacol..

[72] Xie Z., Xie J. (2005). The Na/K-ATPase-mediated signal transduction as a target for new drug development. Front Biosci..

[73] Cantor R.S. (2002). Size distribution of barrel-stave aggregates of membrane peptides: Influence of the bilayer lateral pressure profile. Biophys. J..

[74] Suzuki K., Oda Y., Oda K., Tanaka S., Sakaino Y., Matsudaira T. (2004). Non-genomic action of 17-beta-estradiol on opening of Ca^2+^- and voltage-activated K^+^ channel in lacrimal acinar cells. Tokai J. Exp. Clin. Med..

[75] Coiret G., Matifat F., Hague F., Ouadid-Ahidouch H. (2005). 17-beta-estradiol activates maxi-K channels through a non-genomic pathway in human breast cancer cells. FEBS Lett..

[76] Zhanping W., Xiaoyu P., Na C., Shenglan W., Bo W. (2007). Voltage-gated K+ channels are associated with cell proliferation and cell cycle of ovarian cancer cell. Gynecol. Oncol..

[77] Klimatcheva E., Wonderlin W.F. (1999). An ATP-sensitive K^+^ current that regulates progression through early G1 phase of the cell cycle in MCF-7 human breast cancer cells. J. Membr. Biol..

[78] Monen S.H., Schmidt P.H., Wondergem R. (1998). Membrane potassium channels and human bladder tumor cells. I. Electrical properties. J. Membr. Biol..

[79] Gavrilova-Ruch O., Schonherr K., Gessner G., Schonherr R., Klapperstuck T., Wohlrab W., Heinemann S.H. (2002). Effects of imipramine on ion channels and proliferation of IGR1 melanoma cells. J. Membr. Biol..

[80] Preussat K., Beetz C., Schrey M., Kraft R., Wolfl S., Kalff R., Patt S. (2003). Expression of voltage-gated potassium channels Kv1.3 and Kv1.5 in human gliomas. Neurosci. Lett..

[81] Abdul M., Santo A., Hoosein N. (2003). Activity of potassium channel-blockers in breast cancer. Anticancer Res..

[82] Marino A.A., Iliev I.G., Schwalke M.A., Gonzalez E., Marler K.C., Flanagan C.A. (1994). Association between cell membrane potential and breast cancer. Tumour Biol..

[83] Nilius B., Wohlrab W. (1992). Potassium channels and regulation of proliferation of human melanoma cells. J. Physiol..

[84] Lee Y.S., Sayeed M.M., Wurster R.D. (1993). Inhibition of cell growth by K^+^ channel modulators is due to interference with agonist-induced Ca^2+^ release. Cell Signal..

[85] Peppelenbosch M.P., Tertoolen L.G., de Laat S.W. (1991). Epidermal growth factor-activated calcium and potassium channels. J. Biol. Chem..

[86] Johnson R.M., Harrison S.D., Maclean D. (2011). Therapeutic applications of cell-penetrating peptides. Methods Mol. Biol..

[87] Nekhotiaeva N., Elmquist A., Rajarao G.K., Hallbrink M., Langel U., Good L. (2004). Cell entry and antimicrobial properties of eukaryotic cell-penetrating peptides. FASEB J..

[88] Kol M.A., de Kruijff B., de Kroon A.I. (2002). Phospholipid flip-flop in biogenic membranes: What is needed to connect opposite sides. Semin. Cell Dev. Biol..

[89] May L.T., Leach K., Sexton P.M., Christopoulos A. (2007). Allosteric modulation of G protein-coupled receptors. Annu. Rev. Pharmacol. Toxicol..

[90] Conn P.J., Christopoulos A., Lindsley C.W. (2009). Allosteric modulators of GPCRs: A novel approach for the treatment of CNS disorders. Nat. Rev. Drug Dis..

[91] Hamza A., Sarma M.H., Sarma R.H. (2003). Plausible interaction of an alpha-fetoprotein cyclopeptide with the G-protein-coupled receptor model GPR30: Docking study by molecular dynamics simulated annealing. J. Biomol. Struct. Dyn..

[92] Mizejewski G.J. (1995). Phylogeny of alpha-fetoprotein in vertebrates: Survey of biochemical and physiological data. Crit. Rev. Eukaryot. Gene Expr..

[93] Butterstein G., Mizejewski G.J. (1999). Thyroid hormone induction of frog metamorphosis: Regulation by mammalian alpha-fetoprotein. Comp. Biochem. Physiol..

[94] Mizejewski G.J., Butterstein G. Influence of mammalian alpha-fetoprotein on frog metamorphosis.

[95] Richardson B.E., Mizejewski G.J. Pregnancy and subsequent breast cancer risk: Involvement of alpha-fetoprotein.

[96] Lodge M., Smith G.W., Mizejewski G.J. The effect of alpha-fetoprotein peptide on microtubule polymerization.

[97] Mizejewski G.J., Dauphinee M.J. An alpha-fetoprotein-derived peptide can inhibit growth and induce cytostasis in multiple tumor type both *in vitro* and *in vivo*.

[98] Hua S.C., Chen S.Y., Lu C.H., Kao Y.T., Yu H.I., Chen P.T., Lee Y.R., Chang T.C. (2010). The effects of growth inhibitory peptide on follicular thyroid cancer cell growth, migration, and invasion. Tumori.

[99] Mizejewski G.J., Keenan J.F., Setty R.P. (1990). Separation of the estrogen-activated growth regulatory forms of alpha-fetoprotein in mouse amniotic fluid. Biol. Reprod..

[100] Mizejewski G.J. (1993). An apparent dimerization motif in the third domain of alpha-fetoprotein: Molecular mimicry of the steroid/thyroid nuclear receptor superfamily. BioEssays.

[101] Mizejewski G. (1997). Growth inhibitory peptides.

[102] Mizejewski G. (1998). Methods of using growth inhibitory peptides.

